# HDAC2 attenuates TRAIL-induced apoptosis of pancreatic cancer cells

**DOI:** 10.1186/1476-4598-9-80

**Published:** 2010-04-16

**Authors:** Susanne Schüler, Petra Fritsche, Sandra Diersch, Alexander Arlt, Roland M Schmid, Dieter Saur, Günter Schneider

**Affiliations:** 1 Technische Universität München, Klinikum rechts der Isar, II. Medizinische Klinik, Ismaninger Str. 22, 81675 Munich, Germany; 2 Christian-Albrechts-Universität Kiel, Department of Internal Medicine, University-Hospital Schleswig-Holstein (UKSH)-Campus Kiel, Schittenhelmstr. 12, 24105 Kiel, Germany

## Abstract

**Background:**

Pancreatic ductal adenocarcinoma (PDAC) is one of the most malignant tumors with a dismal prognosis and no effective conservative therapeutic strategies. Although it is demonstrated that histone deacetylases (HDACs), especially the class I HDACs HDAC1, 2 and 3 are highly expressed in this disease, little is known about HDAC isoenzyme specific functions.

**Results:**

Depletion of HDAC2, but not HDAC1, in the pancreatic cancer cell lines MiaPaCa2 and Panc1 resulted in a marked sensitization towards the tumor necrosis factor-related apoptosis-inducing ligand (TRAIL). Correspondingly, the more class I selective HDAC inhibitor (HDACI) valproic acid (VPA) synergized with TRAIL to induce apoptosis of MiaPaCa2 and Panc1 cells. At the molecular level, an increased expression of the TRAIL receptor 1 (DR5), accelerated processing of caspase 8, pronounced cleavage of the BH3-only protein Bid, and increased effector caspase activation was observed in HDAC2-depleted and TRAIL-treated MiaPaCa2 cells.

**Conclusions:**

Our data characterize a novel HDAC2 function in PDAC cells and point to a strategy to overcome TRAIL resistance of PDAC cells, a prerequisite to succeed with a TRAIL targeted therapy in clinical settings.

## Background

The incidence of pancreatic ductal adenocarcinoma (PDAC) is only about 10 in 10^5^, but it is the fourth leading cause of cancer-related death with a 5-year survival rate beyond 5% [1]. As there is no significant improvement in patient survival over the last decades [2] and biologicals like the epidermal growth factor receptor (EGFR) inhibitor erlotinib are only active in a subset of patients [3], there is a need to develop new rational based therapeutic strategies in preclinical settings.

Histone deacetylases (HDACs) deacetylate the ε-amino group of lysines located at the N-terminal tail of histones, which leads to a repressive chromatin formation (heterochromatin) and the suppression of gene expression [4]. In addition to the condensation of chromatin, HDACs deacetylate various proteins to regulate their function. Many of these proteins are transcription factors, such as p53, C/EBPβ, NF-κB and STATs. Therefore changes in the transcriptome upon HDAC inhibitor (HDACI) treatment can be due to a direct modulation of the "histone code" or the consequence of a rather indirect modulation of signaling pathways and transcription factor activities [5-7]. The eighteen deacetylases encoded in the mammalian genome are grouped into class I (HDAC 1, 2, 3 and 8), class II (HDAC 4, 5, 6, 7, 9 and 10), class III (SIRT 1-7) and class IV (HDAC11) enzymes [4]. In tumors, HDACs are involved in the regulation of proliferation, apoptosis, differentiation, migration and angiogenesis [8] and are hence promising targets for therapeutic intervention. In PDAC, the contribution of HDACs towards the control of proliferation, apoptosis and metastasis is clearly documented [9]. Consistently, various HDACI were developed over the last years and are now tested in numerous clinical trials [10]. However, HDACI as monotherapeutics are only effective in a defined subset of hematological tumors and there are several evidences that rational- and molecular-defined HDACI-based combination therapies are more useful for the treatment of solid cancers [11]. Defining suitable HDACI-based combinations is especially important in PDAC since a recent phase II clinical trial failed to demonstrate effectiveness of the weak HDACI CI-994 combined with the current standard chemotherapeutic gemcitabine [12].

In this study we show that specific depletion of HDAC2, but not HDAC1, sensitizes PDAC cells towards tumor necrosis factor-related apoptosis-inducing ligand (TRAIL)-induced apoptosis, suggesting a new therapeutic strategy.

## Results

### HDAC2 depletion sensitizes PDAC cells towards TRAIL-induced apoptosis

**Figure 1:**
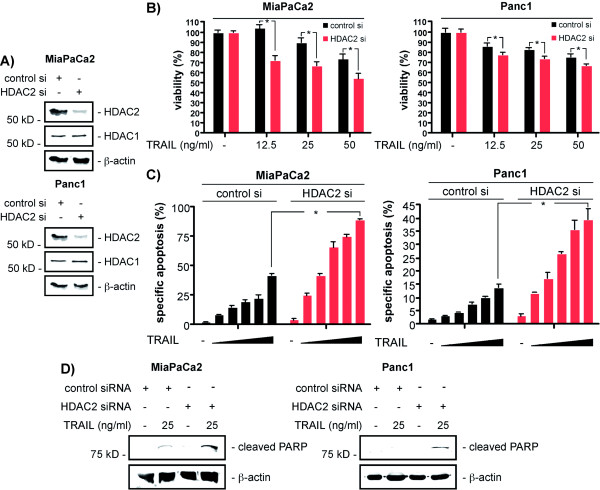
**HDAC2 depletion sensitizes PDAC cells towardsTRAIL**. **A) **Western blot analysis of HDAC2 and HDAC1 48 hours after the transfection of MiaPaCa2 (upper panel) and Panc1 cells (lower panel) with a control siRNA or a HDAC2-specific siRNA. β-actin controls equal protein loading. **B) **MiaPaCa2 (left graph) and Panc1 cells (right graph) were transfected with a control siRNA or a HDAC2-specific siRNA. 48 hours after the transfection the cells were treated with increasing doses of TRAIL as indicated for additional 24 hours or left as an untreated control. Viability was determined using MTT assays (Student's t-test: * p < 0.05 versus controls). **C) **MiaPaCa2 (left graph) and Panc1 cells (right graph) were transfected with a control siRNA or a HDAC2-specific siRNA. 48 hours after transfection the cells were treated with increasing doses of TRAIL (6.25, 12.5, 25, 50 and 100 ng/ml) for additional 24 hours or left as an untreated control. Apoptotic cells were quantified by fluorescence microscopy after Hoechst staining (Student's t-test: * p < 0.05 versus controls). **D) **MiaPaCa2 and Panc1 cells were transfected with a control siRNA or a HDAC2-specific siRNA. 48 hours after the transfection the cells were treated with 25 ng/ml TRAIL for additional 24 hours or left as an untreated control. Cleaved PARP western blotting was used as an indirect measurement of caspase activity. β-actin controls equal protein loading.

We recently observed the HDAC2 mediated control of the DNA-damage response in PDAC cells [13]. To investigate HDAC2 function in the extrinsic apoptotic pathway, we used HDAC2-specific siRNA in PDAC cells (figure 1A). As shown in figure 1B, HDAC2-depleted MiaPaCa2 and Panc1 cells revealed a distinctly decreased viability after the treatment with TRAIL as compared to control siRNA transfected cells. Consistently, the TRAIL-induced apoptotic fraction was significantly increased in a dose-dependent manner in HDAC2-depleted MiaPaCa2 and Panc1 cells (figure 1C). Increased apoptosis induction by TRAIL in MiaPaCa2 and Panc1 cells was further validated using western blots for cleaved PARP (figure 1D). In addition, increased PARP cleavage in HDAC2 siRNA transfected DanG and BxPc3 cells was observed, arguing for a general control of extrinsic apoptotic signaling by HDAC2 in PDAC cells (data not shown).

### Valproic acid sensitizes PDAC cells towards TRAIL-induced apoptosis

**Figure 2:**
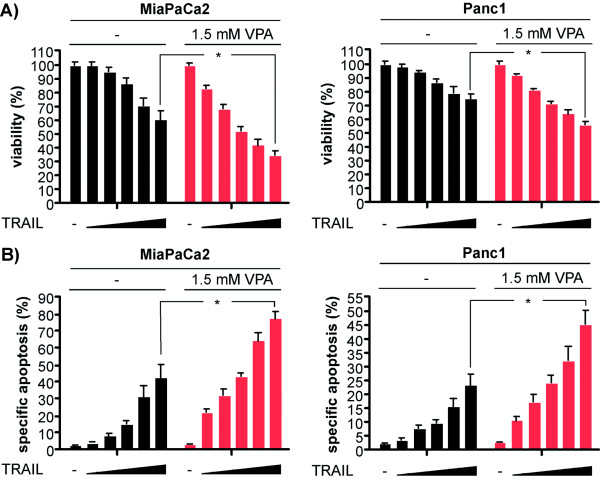
**The HDACI VPA sensitizes PDAC cells towards TRAIL-induced apoptosis**. **A) **MiaPaCa2 (left graph) and Panc1 cells (right graph) were pre-treated with VPA or were left as an untreated control. After 48 hours the cells were treated with TRAIL or the combination of TRAIL and VPA for additional 24 hours or were left as an untreated control. TRAIL was used at concentrations of 6.25, 12.5, 25, 50 and 100 ng/ml. Viability was measured by MTT assays (Student's t-test: * p < 0.05 versus controls). **B) **MiaPaCa2 (left graph) and Panc1 cells (right graph) were pre-treated with VPA or were left as an untreated control. After 48 hours the cells were treated with TRAIL or the combination of TRAIL and VPA for additional 24 hours or were left as an untreated control. TRAIL was used at concentrations of 6.25, 12.5, 25, 50 and 100 ng/ml. Apoptotic cells were quantified by fluorescence microscopy after Hoechst staining (Student's t-test: * p < 0.05 versus controls).

Since valproic acid (VPA) is a more class I specific HDACI and known to deplete HDAC2 via a proteasomal pathway [14,15], we validated the results obtained with functional genomics using VPA. We used VPA at a concentration of 1.5 mM, which is achievable in therapeutical settings and has no influence on PDAC cell proliferation or viability [13]. Co-treatment of MiaPaCa2 and Panc1 cells with TRAIL and VPA lead to a significantly reduced viability in a dose-dependent fashion, compared to cells treated with TRAIL alone (figure 2A). In line, an increased apoptotic fraction was observed in VPA and TRAIL co-treated MiaPaCa2 and Panc1 cells (figure 2B), reproducing the results obtained with RNA interference (RNAi) and suggesting that HDAC2 attenuates TRAIL-induced apoptosis in PDAC cells.

### TRAIL mediated apoptosis is not influenced by HDAC1 in PDAC cells

**Figure 3:**
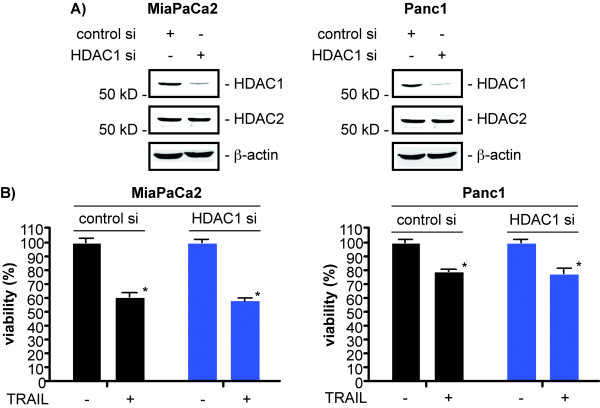
**HDAC1 is not involved in the regulation of TRAIL-induced apoptosis in PDAC cells**. **A) **Western blot analysis of HDAC1 and HDAC2 48 hours after the transfection of MiaPaCa2 (left panel) and Panc1 cells (right panel) with a control siRNA or a HDAC1-specific siRNA. β-actin controls equal protein loading. **B) **MiaPaCa2 (left graph) and Panc1 cells (right graph) were transfected with a control siRNA or a HDAC1-specific siRNA. 48 hours after the transfection the cells were treated with 50 ng/ml TRAIL for additional 24 hours or left as an untreated control. Viability was determined using MTT assays (Student's t-test: * p < 0.05 versus controls).

Since HDAC-dependent non-redundant functions are ill defined, we tested whether HDAC1-depletion also sensitizes for TRAIL-induced apoptotsis. Transfection of HDAC1 siRNA into MiaPaCa2 and Panc1 cells resulted in a specific knockdown of HDAC1, whereas HDAC2 expression was not affected (figure 3A). Nevertheless, TRAIL-induced loss of viability of MiaPaCa2 and Panc1 cells was not changed in HDAC1 siRNA transfected cells compared to control siRNA transfected cells (figure 3B). Furthermore, no increase in the apoptotic fraction was observed in HDAC1-depleted and TRAIL-treated MiaPaCa2 and Panc1 cells in comparison to control siRNA transfected cells (data not shown). This argues that HDAC2 is specifically involved in the regulation of TRAIL induced apoptosis, independently of HDAC1.

### HDAC2-dependent regulation of the BH3-only protein NOXA is not involved in HDAC2-depletion mediated TRAIL sensitization

**Figure 4:**
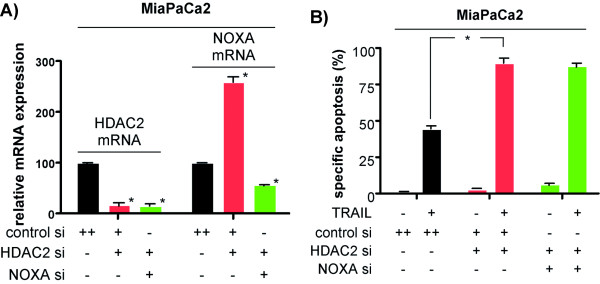
**HDAC2-dependent regulation of NOXA does not contribute to TRAIL sensitization**. **A) **Quantitative NOXA and HDAC2 mRNA expression analysis in MiaPaCa2 cells after the transfection of a control, a HDAC2-specific or the combination of HDAC2- and NOXA-specific siRNAs. Total RNA was prepared 48 hours post-transfection. NOXA and HDAC2 mRNA levels were quantified using real-time PCR analysis and normalized to cyclophilin expression levels (Student's t-test: * p < 0.05 versus controls). **B) **MiaPaCa2 cells were transfected with a control siRNA, a HDAC2-specific siRNA or co-transfected with a HDAC2- and NOXA-specific siRNA as indicated. 48 hours after the transfection the cells were treated with 30 ng/ml TRAIL for additional 24 hours or left as an untreated control. Apoptotic cells were quantified by fluorescence microscopy after Hoechst staining (Student's t-test: * p < 0.05 versus controls).

Recently we observed that depletion of HDAC2 results in an increased expression of the pro-apoptotic BH3-only protein NOXA in PDAC cells [13]. To investigate the influence of NOXA on the HDAC2-dependent sensitization towards TRAIL-induced apoptosis, we simultaneously transfected HDAC2- and NOXA-specific siRNAs into MiaPaCa2 cells. As shown in figure 4A, upregulation of NOXA mRNA by the depletion of HDAC2 was significantly inhibited, when the cells were transfected with both siRNAs. Furthermore, HDAC2-depletion mediated sensitization of MiaPaCa2 cells towards TRAIL-induced apoptosis was not changed in HDAC2 and NOXA siRNA co-transfected cells (figure 4B), demonstrating that HDAC2-mediated sensitization towards death receptor induced apoptosis is independent of NOXA.

### HDAC2 regulates the TRAIL receptor DR5 in MiaPaCa2 cells

**Figure 5:**
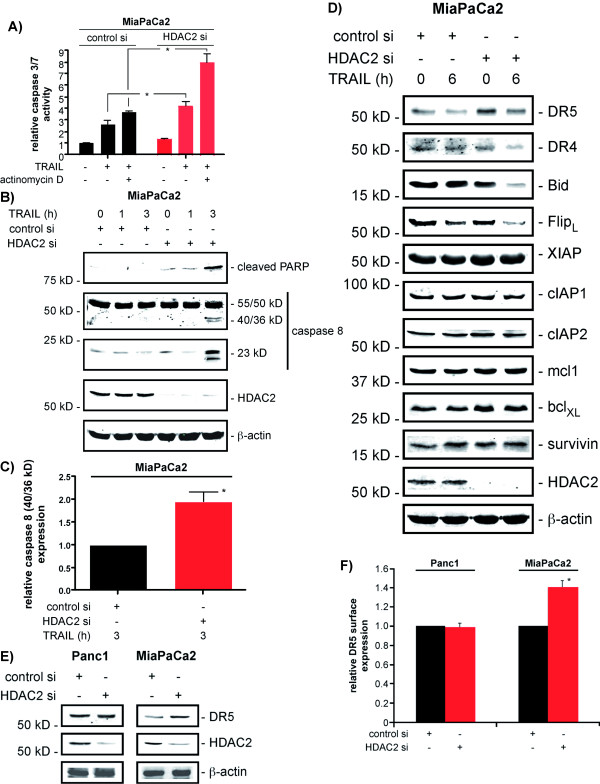
**HDAC2-dependent regulation of DR5 in MiaPaCa2 cells**. MiaPaCa2 cells were transfected with a control or a HDAC2-specific siRNA as indicated. **A) **48 hours after the transfection the cells were treated with 15 ng/ml TRAIL or a combination of TRAIL and actinomycin D (1 μg/ml) for additional 2 hours. Afterwards caspase 3/7 activity was measured (Student's t-test: * p < 0.05 versus controls). **B) **48 hours after the transfection the cells were treated with 30 ng/ml TRAIL as indicated. Western blot determines caspase 8 (55/50 kD), caspase 8 cleavage products (migrating at 40/36 kD and 23 kD), cleaved PARP and HDAC2 expression. β-actin controls equal protein loading. **C) **MiaPaCa2 cells were treated as in B). The caspase 8 cleavage product migrating at 40/36 kD was quantified in control and HDAC2 siRNA transfected MiaPaCa2 cells 3 hours after the TRAIL treatment in four independent experiments (Student's t-test: * p < 0.05 versus controls). **D) **48 hours after the transfection the cells were treated with 30 ng/ml TRAIL for 6 hours. Western blot determines DR5, DR4, Bid, c-Flip, XIAP, cIAP1, cIAP2, mcl1, bcl_XL_, survivin and HDAC2 expression. β-actin controls equal protein loading. **E) **Panc1 (left graph) and MiaPaCa2 cells (right graph) were transfected as indicated. Western blot determines DR5 and HDAC2 expression 48 hours after the transfection. β-actin controls equal protein loading. **F) **Panc1 (left graph) and MiaPaCa2 cells (right graph) were transfected as indicated. 48 hours after the transfection DR5 surface expression was determined by FACS analysis (Student's t-test: * p < 0.05 versus controls).

Recent evidence suggests that HDACs can alter gene expression by the direct deacetylation of ε-amino-lysin groups at the N-terminal tails of histones and/or by the direct influence on various signaling pathways [6]. To discriminate between both possibilities we transfected MiaPaCa2 cells with a control and a HDAC2-specific siRNA and treated the cells with TRAIL or a combination of TRAIL and actinomycin D to inhibit TRAIL signaling-induced transcription. As shown in figure 5A, TRAIL-induced caspase 3/7 activation was significantly increased in HDAC2-depleted MiaPaCa2 cells. Furthermore, blocking transcription in control and HDAC2 siRNA transfected MiaPaCa2 cells resulted in an increased caspase 3/7 activity, arguing that the TRAIL-induced transcription functions mainly to restrain death-receptor-induced apoptosis. Nonetheless, caspase 3/7 activity was significantly higher in TRAIL and actinomycin D co-treated HDAC2-depleted MiaPaCa2 cells than in TRAIL and actinomycin D co-treated control siRNA transfected cells, indicating that a change in the gene expression after HDAC2 depletion or a mechanism not requiring transcription, independently of TRAIL-induced transcription, at least contributes to the increased sensitivity of HDAC2-depleted MiaPaCa2 cells to TRAIL. In line with the increased activity of caspase 3/7 we observed an accelerated cleavage of caspase 8 in HDAC2-depleted and TRAIL-treated MiaPaCa2 cells, which correlated with the appearance of cleaved PARP as an indirect measurement of effector caspase activity (figure 5B and 5C). Similar results were observed in VPA treated MiaPaCa2 cells (data not shown). Furthermore, we detected a distinctly increased cleavage of Bid in HDAC2-depleted and TRAIL-treated MiaPaCa2 cells, compared to control siRNA transfected cells (figure 5D). In transcriptome profiles of HDAC2-depleted PDAC cells, which we have recently published [13], the TRAIL receptor DR5 was 2.2 fold upregulated in MiaPaCa2 cells and marginally increased in Panc1 cells. As shown in figure 5D and 5E, increased DR5 protein expression was detected in HDAC2-depleted MiaPaCa2 cells. In contrast, no change in the expression of DR4, c-Flip, XIAP, cIAP1, cIAP2, mcl1 or survivin was observed in HDAC2-depleted MiaPaCa2 cells (figure 5D). A slight but consistent upregulation of bcl_XL _was observed in HDAC2-depleted MiaPaCa2 cells (figure 5D). Fitting to an increase in apoptosis induced by TRAIL in HDAC2-depleted MiaPaCa2 cells, we observed a more pronounced elimination of c-Flip after the TRAIL treatment in HDAC2 siRNA transfected MiaPaCa2 cells (figure 5D). In contrast to MiaPaCa2 cells, upregulation of DR5 protein expression after HDAC2 depletion was not observed in Panc1 cells (figure 5E). Consistently, DR5 cell surface expression was slightly increased in MiaPaCa2 cells, but not in Panc1 cells (figure 5F). Together, these data argue that HDAC2-dependent sensitization towards TRAIL-induced apoptosis works upstream of Bid in MiaPaCa2 cells.

## Discussion

Deregulation of apoptosis is a hallmark of cancer and is an important cause of therapeutic failure of conventional chemotherapeutics [16,17]. Apoptosis can be initiated by the extrinsic death receptor-dependent as well as the intrinsic mitochondrial pathway and various mechanisms of resistance working on several levels were described in PDAC [18,19]. Triggering of death-receptors, like the CD95 receptor or TRAIL receptors, results in the activation of the initiator caspase 8 via the death-inducing signaling complex (DISC) with subsequent activation of effector caspases. In PDAC cells, which are type II cells, active caspase 8 cleaves the BH3-only protein Bid, which augments the activation of the caspase cascade via the mitochondrium [20,21]. Due to their selective toxicity towards cancer cells, TRAIL receptor agonists are promising cancer therapeutics and currently tested in clinical trials [21]. Furthermore, the observation that high TRAIL expression was correlated with an increased apoptotic index in the human pancreas argues that a TRAIL-based therapy might be a feasible strategy for the treatment of PDAC [22,23]. However, human primary tumor cells often resist TRAIL-induced apoptosis and PDAC cells reveal a relatively high half maximal inhibitor concentration (IC50) for TRAIL although they express the TRAIL receptors DR4 (TRAIL-R1) and DR5 (TRAIL-R2) as well as relevant mediators of the TRAIL receptor signaling pathway [24-26]. Therefore, strategies to sensitize PDAC cells towards TRAIL and to counteract resistance mechanisms are of value for defining new therapies for the treatment of PDAC.

In this study we show that the more class I selective HDACI VPA sensitizes PDAC cells towards TRAIL-induced apoptosis. Furthermore, we demonstrate that HDAC2, which is highly expressed in human PDACs [13,27], controls resistance towards TRAIL. These results in combination with the high expression of HDAC2 in many solid tumors [28] and the recent observation of HDAC2-dependent sensitization of breast and pancreatic cancer cells towards topoisomerase II inhibitors [13,29], of breast cancer cells towards antihormonal therapy [30] and of colon cancer cells towards TNFα [31], characterizes HDAC2 as an important therapeutic target. Furthermore, HDAC2 contributes to EMT, one initial mechanism of metastasis, by the downregulation of E-cadherin in pancreatic cancer cells [32]. Altogether, these data may base the development of HDAC2 isoenzyme specific inhibitors with increased inhibitory capacity and lower toxicity compared to current HDACI [33].

Several TRAIL resistance mechanisms working at the level of the TRAIL receptors, the TRAIL DISC, the inhibitor of apoptosis proteins (IAPs) or the mitochondrium were described in PDAC cells [18,19]. However, consistent with our recent transcriptome profiling [13], we were not able to detect a clear downregulation of c-Flip, XIAP, cIAP1, cIAP2, mcl1, bcl_XL _or survivin at the protein level after depletion of HDAC2, although an involvement of these proteins in the control of TRAIL resistance was described [26,34-43]. Using the HDACI butyrate, Natoni et al. described a HDAC-dependent control of sensitivity of PDAC cells towards Fas-induced apoptosis. At the molecular level the authors observed a reduced expression of bcl_XL _and c-Flip after the treatment with butyrate [44]. The failure to detect downregulation of bcl_XL _and c-Flip after the sole HDAC2 depletion points to compensatory mechanisms working to maintain expression of these genes in PDAC cells. Nonetheless, our results are consistent with recent observations in HCT116 colon cancer cells, where HDACI treatment or depletion of HDAC2, but not HDAC1, sensitizes towards TNFα-induced apoptosis [31]. Here, a contribution of NF-κB in the HDAC2-dependent sensitization was proposed, since HDAC2 siRNAs reduce the TNFα mediated activation of a NF-κB luciferase reporter gene [31]. Consistently, a recent study reveals that treatment of the PDAC cell line Panc1 with the HDACI SAHA reduces basal binding of RelA to a consensus κB oligonucleotide [27]. Alike, we observed dependency of the basal NF-κB transcriptional activity on the presence of HDAC2 in PDAC cells (data not shown). The observation that basal, but not induced, NF-κB activity contributes to TRAIL resistance of PDAC cells [45] and that inhibition of NF-κB by the super-inhibitor delta-N-IκBα [46] or a p65 siRNA [47] resulted in profound sensitization towards TRAIL-induced apoptosis in PDAC cells, might point to the contribution of NF-κB in our model system. However, we cannot rule out NF-κB-independent mechanisms at the moment and the ultimate clarification awaits further experiments beyond the scope of the article.

HDACI sensitize cancer cells towards TRAIL and several mechanisms, like upregulation of TRAIL and TRAIL receptors, downregulation of c-Flip, XIAP or members of the anti-apoptotic bcl2 family members as well as the modulation of NF-κB activity were shown to contribute [48,49]. As observed in many studies using HDACI to sensitize towards TRAIL, we observed a slight upregulation of DR5 protein abundance and cell surface expression in MiaPaCa2 cells after the depletion of HDAC2. The notice that HDACI-dependent TRAIL sensitization is independent of DR5 [50] and that DR5 regulation was not observed in Panc1 cells points to an alternative HDAC2 regulated mechanism contributing to the sensitization of PDAC cells. The demonstration of accelerated TRAIL-induced Bid cleavage after the depletion of HDAC2 argues that an event upstream of Bid is under HDAC2 control in PDAC cells. Accordingly, HDACI-dependent increased recruitment of the DISC component Fas-associated death domain protein (FADD) to the TRAIL-R1 was recently shown to play a role in the HDACI-mediated sensitization of CLL cells to TRAIL [51]. Although accelerated DISC formation and function after HDAC2 depletion is an attractive possibility, it is currently not known whether HDAC2 can control DISC formation and proof of this alternative explanation awaits additional experiments.

## Conclusions

In summary, we have described a novel non-redundant HDAC2 function in PDAC cells that provides the rationale for further preclinical evaluation of a HDACI (HDAC2 isoenzyme-specific) and TRAIL combination therapy. Furthermore, our experiments point to a way to overcome TRAIL resistance of PDAC cells, needed to succeed with a TRAIL targeted therapy in clinical settings.

## Methods

### Cell culture, siRNA transfection and reagents

The pancreatic cancer cell lines MiaPaCa2 and Panc1 were cultivated as recently described [13]. Valproic acid (VPA) and TRAIL were purchased from EMD (EMD Biosciences, San Diego, CA, USA). Actinomycin D was from Sigma-Aldrich (Sigma-Aldrich, Munich, Germany). Untreated controls received vehicle alone. Double-stranded siRNAs were transfected at a final concentration of 50 nM using oligofectamine (Invitrogen, Karlsruhe, Germany) according to the manufacturer's protocol. siRNAs were purchased from Eurofins, Ebersberg, Germany. Sequences of the used siRNAs were: control siRNA 5' C A G T C G C G T T T G C G A C T G G dtdt 3', HDAC2 siRNA 5' G C C T C A T A G A A T C C G C A T G dtdt 3 ', HDAC1 siRNA 5' G C A G A T G C A G A G A T T C A A C dtdt 3', NOXA siRNA 5' G G A A G T C G A G T G T G C T A C T dtdt 3'. For the simultaneous transfection of siRNAs directed against two different genes, the total amount of siRNA (100 nM) was kept constant using control siRNA.

### Statistical methods

All data were obtained from at least three independent experiments performed in triplicate, and the results are presented as mean and standard error of the mean (S.E.M.). To demonstrate statistical significance a two-tailed Student's t-test was used. p-values are indicated and * denotes a p-value of at least < 0.05.

### Quantitative Reverse-Transcriptase PCR

Total RNA was isolated from pancreatic carcinoma cell lines using the RNeasy kit (Qiagen, Hilden, Germany) following the manufacturer's instructions. Quantitative mRNA analyses were performed as previously described using real-time PCR analysis (TaqMan, PE Applied Biosystems, Norwalk CT) [52,53]. Expression levels were normalized using cyclophilin. Primer sequences were as follows: cyclophilin-fw 5' A T G G T C A A C C C C A C C G T G T 3'; cyclophilin-rev 5' T C T G C T G T C T T T G G G A C C T T G T C 3'; HDAC2-fw 5' A G C A T C A G G A T T C T G T T A C G T T A A T G A 3'; HDAC2-rv 5' C A A C A C C A T C A C C A T G A T G A A T A T C T 3'; NOXA-fw 5' C G G A G A T G C C T G G G A A G A A 3'; NOXA-rev 5' C C A A A T C T C C T G A G T T G A G T A G C A 3'.

### Total cell lysates and Western blot

Whole cell lysates were prepared and western blots were done as recently described [52,53]. The following antibodies were used: DR4, DR5 (ProScience Inc., Poway, CA, USA); HDAC2 (H-54, sc-7899) (Santa Cruz Biotechnology, Santa Cruz, CA, USA); XIAP, cIAP1, cIAP2, survivin (R&D Systems, Minneapolis, MN, USA); Bid, bcl_XL _(Cell Signaling Technology Inc., Danvers, MA, USA); NOXA, mcl1 (Alexis Biochemicals, San Diego, CA, USA); c-Flip, cleaved PARP, caspase 8 (BD Biosciences, Heidelberg, Germany); HDAC1 (Upstate/Millipore, Billerica, MA, USA); β-actin (Sigma-Aldrich, Munich, Germany). One representative western blot out of at least three independent experiments is shown. Western blots were quantified using Odyssey Infrared Imaging System (LI-COR Biosciences, Bad Homburg, Germany), assuring measurements in the linear range.

### MTT- and Caspase 3/7-assay

Viability of the cells was measured using MTT-assays performed according to the manufacturer's protocol (Roche Applied Science, Mannheim, Germany). Caspase 3/7 activity was determined using Promega's Caspase-Glo 3/7 assay according to the manufacturer's instructions (Promega, Madison, WI, USA).

### Analysis of cell surface expression of DR5

Cells were transfected as indicated. After 48 h cells were trypsinized, washed once with PBS and suspended in PBS containing 2 μg/ml primary antibody (anti-DR5; R&D Systems, Minneapolis, USA) or control IgG. Cells were stained on ice for 60 min, then washed with 3 ml cold PBS and incubated with the secondary antibody (1:100 dilution PE-conjugated goat anti-mouse F(ab')2 anti-IgG+IgM (Jackson ImmunoResearch, Suffolk, England)) for 60 min, on ice, in the dark. After an additional wash with 3 ml cold PBS, cells were suspended in 1 ml cold FACS buffer and 10.000 cells per sample were analyzed using fluorescence flow cytometry (Galaxy Argon Plus, Dako, Glostrup, Denmark) and results were analysed with the FLOMAX software (Dako).

## Competing interests

The authors declare that they have no competing interests.

## Authors' contributions

SS, PF, SD, AA, DS, and GS designed and conducted the experiments. SS, PF, SD, AA, DS, and GS analyzed and interpreted data. SS, PF, SD, AA, DS, RMS and GS drafted the manuscript and revised it critically for important intellectual content. All authors read and approved the final manuscript.
